# Acetone and Toluene Gas Sensing by WO_3_: Focusing on the Selectivity from First Principle Calculations

**DOI:** 10.3390/nano12152696

**Published:** 2022-08-05

**Authors:** Mario Italo Trioni, Fausto Cargnoni, Stefano Americo, Eleonora Pargoletti, Gian Luca Chiarello, Giuseppe Cappelletti

**Affiliations:** 1 National Research Council of Italy, Institute of Chemical Sciences and Technologies “Giulio Natta”, Via Golgi 19, 20133 Milano, Italy; 2 Department of Chemistry, University of Milano, Via Golgi 19, 20133 Milano, Italy

**Keywords:** monoclinic WO_3_, gas sensing, chemiresistor, acetone, toluene, selectivity, Density Functional Theory

## Abstract

Sensitivity and selectivity are the two major parameters that should be optimized in chemiresistive devices with boosted performances towards Volatile Organic Compounds (VOCs). Notwithstanding a plethora of metal oxides/VOCs combinations that have been investigated so far, a close inspection based on theoretical models to provide guidelines to enhance sensors features has been scarcely explored. In this work, we measured experimentally the sensor response of a WO_3_ chemiresistor towards gaseous acetone and toluene, observing a two orders of magnitude higher signal for the former. In order to gain insight on the observed selectivity, Density Functional Theory was then adopted to elucidate how acetone and toluene molecules adsorption may perturb the electronic structure of WO_3_ due to electrostatic interactions with the surface and hybridization with its electronic structure. The results of acetone adsorption suggest the activation of the carbonyl group for reactions, while an overall lower charge redistribution on the surface and the molecule was observed for toluene. This, combined with acetone’s higher binding energy, justifies the difference in the final responses. Notably, the presence of surface oxygen vacancies, characterizing the nanostructure of the oxide, influences the sensing performances.

## 1. Introduction

The growing concerns in reliable detection of gaseous species have led to the development of newly emerged gas sensors for a wide variety of applications, such as environment pollution monitoring, air quality investigating, medical diagnostics, food technology, etc. [1]. Particularly, chemoresistive sensors [2] have attracted the scientific community due to their good response (down to ppb), ease in synthesis, and cost effectiveness. Plenty of nanometric metal oxide semiconductors (MOS) have been tested, including SnO_2_ [3,4], ZnO [5,6], WO_3_ [7,8], NiO [9], In_2_O_3_ [10], MoO_3_ [11], and TiO_2_ [12]. Common problems associated with these materials are the high sensing temperatures and the poor specificity to a target gas. Specifically, in the health care sector, a breath analyzer [13] must selectively sense a specific biomarker in a very complex exhaled matrix. Several methods for solving have been proposed in the recent literature: (i) by modifying the oxides shape and the exposed crystallographic facets, (ii) by adding a noble metal catalyst to a transition metal oxide, (iii) by designing mixed oxides or composite materials, and (iv) by exploiting sensor arrays coupled with principal component analysis (PCA) and eventually with the aid of machine learning algorithms. Choi et al. [14] reported that SnO_2_ nanosheets, which mainly exposed the (101) crystal facet, exhibit a higher gas selectivity towards acetone molecules among several investigated biomarkers, such as hydrogen, isoprene, and toluene. Moreover, the loading of a noble metal catalyst is known to be effective to enhance BTEX detection [15]. Representative BTEX sensing materials could be Pd- and Pt-SnO_2_ nanowires [16], Pt-ZnO nanoparticles [17], Pd-WO_3_ nanocubes [18], Au-ZnO nanopowders [19], Ru-In_2_O_3_ nanosheets [20], etc. Furthermore, in our recent work [21] Sn*_x_*Ti_1−x_O_2_/graphene oxide (GO)-based materials were synthesized via simple hydrothermal method, varying the titanium content in the tin dioxide matrix. A greater selectivity towards acetone analyte (at ppb level, also at room temperature) was obtained at high Ti amount. Instead, solid solutions having a higher content of tin exhibited greater selectivity towards bigger and non-polar molecules (as toluene) at 350 °C, rather than polar ones. Notably, Song et al. [22] proposed an array of Pd/SnO_2_/Al_2_O_3_ sensors with high surface area-to-volume ratio showing enhanced surface interactions with different gases. PCA and support vector machine (SVM) algorithms have been successfully employed for multi-gases (H_2_, formaldehyde, toluene, and acetone) discrimination in different relative humidity. Nevertheless, despite a jungle of experimental observations, the physical origin of the enhanced sensitivity and selectivity of the above-mentioned systems towards polar/apolar gaseous species is still under debate. In this respect, first principles calculations might be of great help to shed light on the electronic perturbations induced by the adsorption of volatile organic compounds onto the surface of selected materials, as well as the charge transfer between gas-phase molecules and the underlying atomic layers. The scenario derived from theoretical models might be a guide for the design and the development of novel gas sensors with enhanced sensitivity and boosted selectivity. Keeping this is mind, we performed Density Functional Theory (DFT)-based computations to derive a rigorous perspective on the origin of the selectivity of WO_3_, a widely exploited gas sensor, towards two common organic molecules with different chemical properties. The role of native defects (i.e., oxygen vacancies) in determining the response of WO_3_ towards adsorption of acetone and toluene is extensively discussed. Based on our data, we also trace a relation between the performance of this material and the location, the concentration, and the structuring of oxygen vacancies within the sensor.

## 2. Materials and Methods

### 2.1. WO_3_ Synthetic Route

The adopted chemicals (by Sigma-Aldrich, Milano, Italy) were of reagent-grade purity; doubly distilled water passed through a MilliQ apparatus was used. 2.0 g of Pluronic^®^ P123 (poly(ethylene oxide)_20_poly(propylene oxide)_70_poly(ethylene oxide)_20_, 5.8 kDa mass weight; as templating agent) were dispersed in 50 mL of MilliQ water at ca. 30 °C under moderate stirring in order to avoid foam formation. Then, 3.25 g of Na_2_WO_4_·2H_2_O were added followed by a dropwise addition of 10 mL of 37% HCl. The system was stirred (200 rpm) at 30 °C for 3 h; afterwards, the obtained sol was dried in the oven at 60 °C and calcined at 400 °C under oxygen flux (5 h, 9 NL h^−1^) to remove the majority of the organic traces. Finally, the powder was repeatedly washed with ethanol and MilliQ water to eliminate the possible templating agent residues. Then, the as-synthesized nanoparticles were dried in oven at 60 °C.

### 2.2. WO_3_ Physicochemical Characterizations

X-ray Powder Diffraction (XRPD), specific surface area (*S*_BET_), and porosity distribution (total pores volume, V_tot. pores_) analyses were performed according to the specifications reported in our previous study [7].

### 2.3. Sensing Tests on WO_3_-Deposited Interdigitated Electrodes (IDEs)

WO_3_ powder was deposited by the hot-spray method on Al_2_O_3_ interdigitated gold electrodes (Au-IDEs), as reported in our previous work [7]. Sensing tests were carried out using a custom-built stainless steel cell already described elsewhere [21]. In particular, the sensor response was measured while flowing a simulated air (80% N_2_–20% O_2_) gas mixture (total flow rate of 0.5 L min^−1^) in the presence of different analytes (acetone or toluene) concentrations. Analytes flow was varied by dilution from a starting 500 ppm concentration (in N_2_ gas mixture), keeping constant the total flow rate. The dynamic response was recorded by an Autolab PGStat30 (Eco Chemie, Utrecht, The Netherlands) potentiostat/galvanostat controlled by NOVA 2.0 software, applying a bias of +1.0 V. Tests were performed at relative humidity lower than 2% and at 300 °C. The sensor signal is reported as (R_air_/R_analyte_) − 1, together with the relative response and recovery times, according to previous literature [6].

### 2.4. Computational Details

*Ab initio* calculations have been performed on the γ-monoclinic phase of WO_3_ using the Density Functional Theory with the GGA Perdew–Burke–Ernzerhof exchange-correlation energy functional [23], as implemented in the SIESTA code [24]. Troullier–Martins pseudopotentials [25] and double-ζ localized atomic orbitals have been used as basis set. The Brillouin zone has been sampled using a 6 × 6 × 6 k-points mesh for the 32 atoms bulk unit cell, and retaining the same k-point density for all other systems considered. Differently, the electronic properties of relaxed systems are computed within the GGA-1/2 approach, applied to the oxygen atoms only (see Refs. [26,27] for more details). This method allows to describe the energy gaps much better than available DFT approaches. GGA-1/2 was chosen because it is completely parameter-free and it does not affect the computational cost of the calculations.

## 3. Results and Discussion

### 3.1. WO_3_ Physicochemical Features

**Figure 1:**
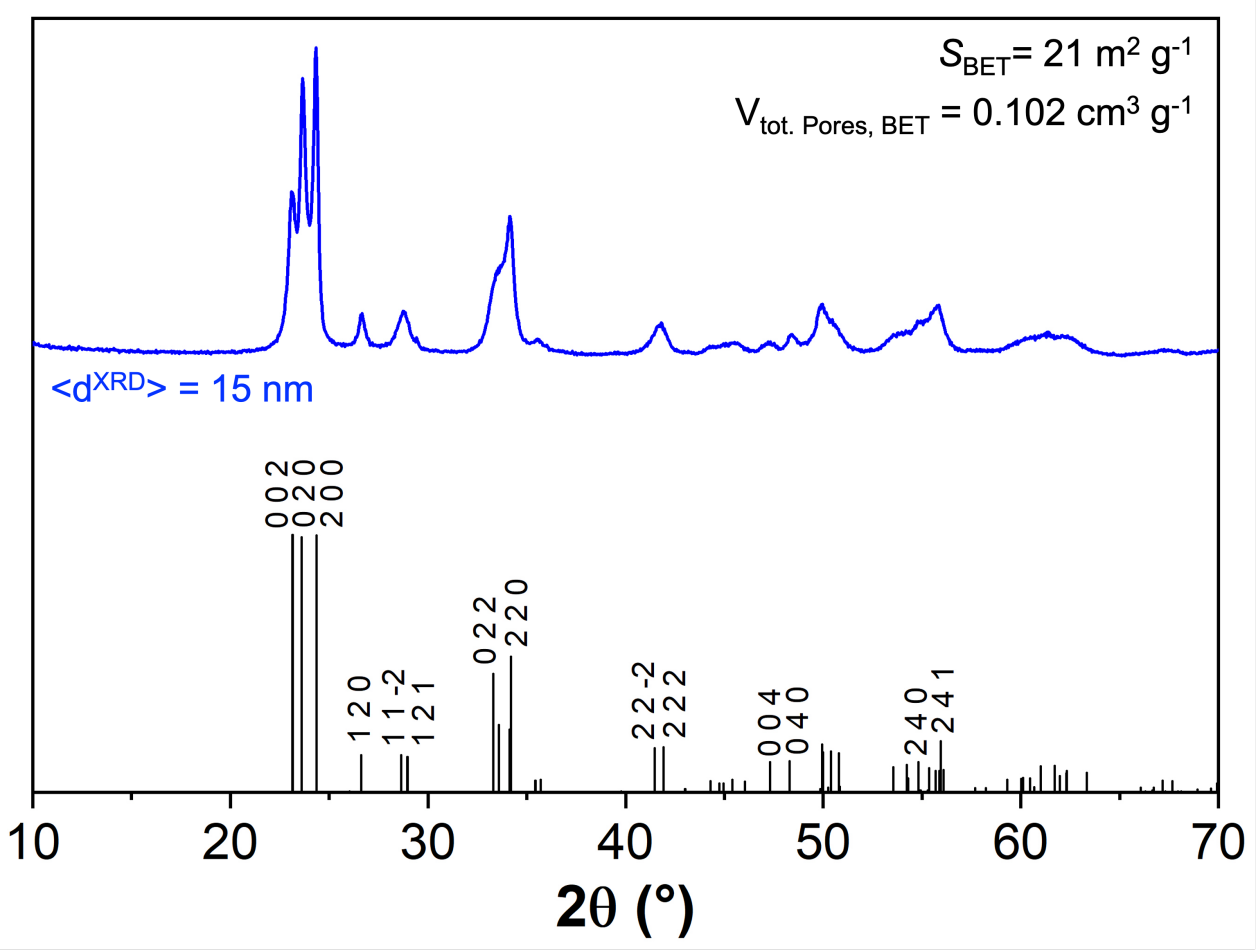
WO_3_ XRPD pattern in which the main peaks are assigned to Miller’s indexes (International Centre for Diffraction Data–ICDD n. 01-075-2072). Average crystallite domains size (<d^XRPD^>), specific surface area (*S*_BET_), and total pores volume (V_tot. pores_) are also evidenced.

Structural and surface properties of the synthesized nanoparticles were investigated by XRPD and BET analyses, respectively. Figure 1 shows the acquired X-ray pattern in which the characteristic peaks at ca. 2θ = 23.1°, 23.7°, and 24.3° are ascribable to the (0 0 2), (0 2 0), and (2 0 0) Miller’s indexes relative to the γ-monoclinic phase. Furthermore, the XRPD spectrum reveals a quite good degree of crystallinity, resulting in crystallite domains the size of about 15 nm. As far as it concerns the specific surface area, the obtained value is relatively low (21 m^2^ g^−1^) as expected for WO_3_, but slightly higher with respect to the one reported in our previous work [7]. Analogously, the total pore volume is greater (around 0.102 cm^3^ g^−1^) probably due to the presence of Pluronic^®^ P123 tenside that may have contributed to an increase in both *S*_BET_ and pores diameter.

### 3.2. WO_3_ Structure

**Figure 2:**
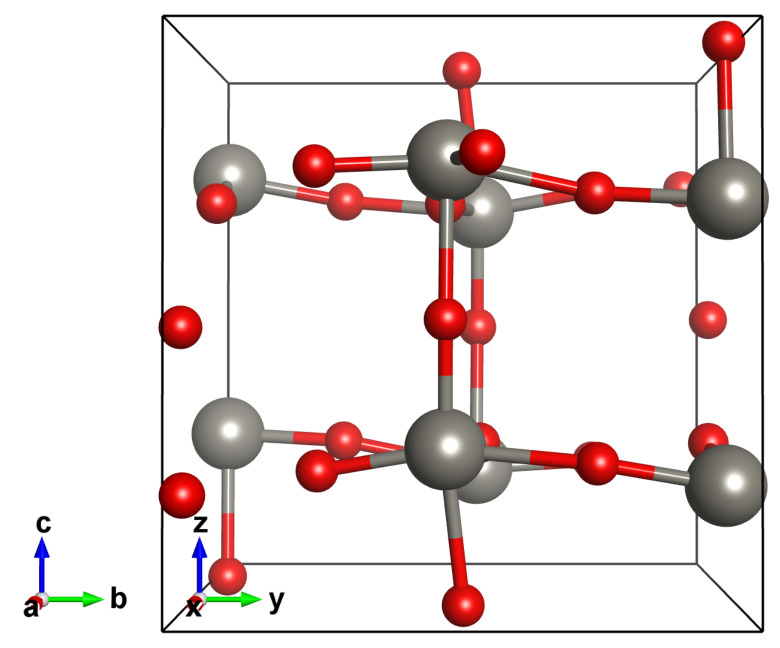
Atomic structure of the γ-monoclinic WO_3_. Tungsten in black and oxygen in red.

The first step in studying the structural, electronic, and magnetic properties of adsorbates on WO_3_ surface is to verify the accuracy of the theoretical and computational frameworks in describing the bulk properties. We considered the γ-monoclinic phase of WO_3_ (P2_1/*n*_) constituted by eight WO_6_ octahedrons jointed via oxygen atoms bridging two tungsten ones (W_8_O_24_). The optimized unit cell (starting from an experimental determination of this phase [28]) provided the following values: 7.46 Å, 7.68 Å, and 7.88 Å for a, b, and c cell vectors, respectively, where b is the unique axis. The angle between a and c is found to be 90.4°. The agreement with experimental data is excellent [28,29,30,31]. The octahedrons are slightly tilted with respect to one another and W atoms are a bit off-center, hence the O-W bond lengths along the three crystallographic vectors differ, as reported in Figure 2.

**Figure 3:**
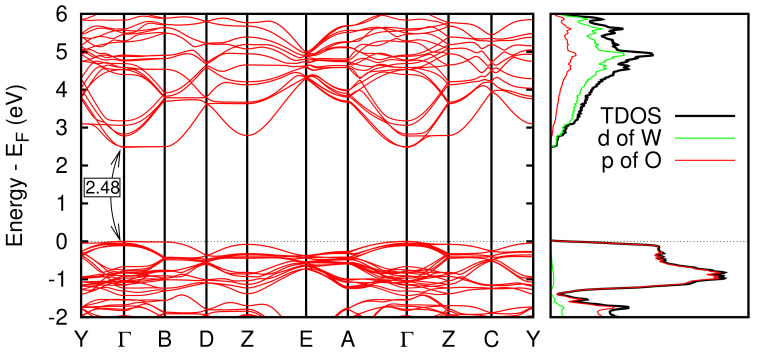
Band structure of γ-monoclinic WO_3_ and density of states.

We obtained a good agreement with experimental data also for what concerns the electronic properties. Indeed, our GGA-1/2 theoretical approach provides a value for the electronic band gap of 2.48 eV, very close to the experimental one (∼2.63 eV) [7,32,33,34,35,36,37], despite the widely used local or semi-local approximations to the exchange-correlation energy functional of DFT typically underestimates significantly this value. Figure 3 shows the band structure and the density of states (DOS) of the γ-monoclinic phase.

**Figure 4:**
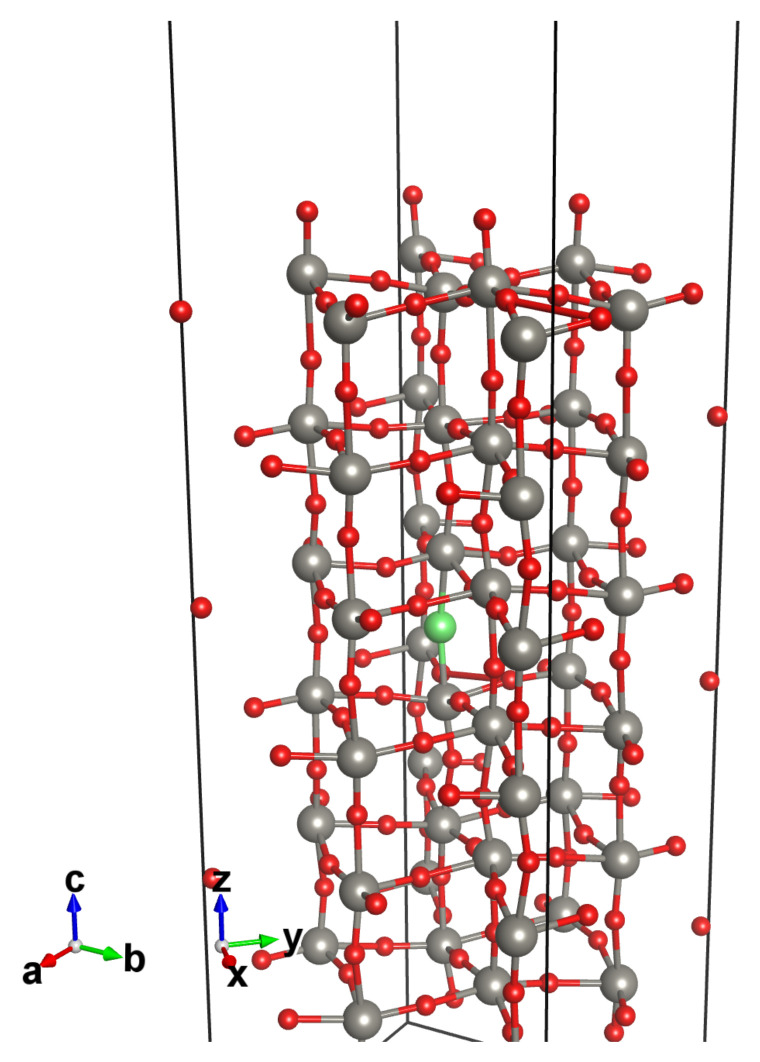
Atomic structure of the γ-monoclinic WO_3_(001) reconstructed surface. W in black and O in red; the bulk vacancy site is indicated in green.

The intrinsic *n*-doped semiconductor behavior commonly attributed to WO_3_ in the literature [38,39,40] is probably due to the most abundant defect found in this material, i.e., oxygen vacancies (V_O_). They would cause the Fermi level to shift towards the conduction band, populating new states well above the valence bands and producing the observed intrinsic *n*-doping. This explains the electric response of WO_3_-based devices. Actually, the standard procedure for producing WO_3_ gives rise to a natural lack of oxygen with respect to the stoichiometric ratio. Hence, we modeled all the WO_3_ surfaces considering the presence of an oxygen vacancy into the bulk. The most stable surface of γ-WO_3_, according to literature [41,42,43], is the (001)c(2 × 2) reconstructed one. This surface is modeled by a slab whose width corresponds to six bulk unit cells (6 × 32 atoms). We relaxed the first four WO_6_ octahedrons layers. The surface reconstruction was obtained by removing half of the exposed oxygen atoms in a chessboard fashion, as can be seen in Figure 4.

### 3.3. Acetone and Toluene Experimental Sensing

As extensively discussed in a recent study by our group [7], the sensing of acetone on WO_3_ is based on a catalytic process where the analyte exchanges oxygen atoms directly with the surface and decomposes to smaller, oxidized compounds, such as CO_2_. Every oxygen atom that detached from the surface releases two electrons into the conduction band of the material, producing a marked decrease in its resistivity and a consequent increase in the measured electric current. The amount of surface oxygen defects at the equilibrium determines the overall peak height. A partial contribution to the overall conductivity is expected to derive also from the direct charge transfer between the surface and the analyte upon adsorption.

**Figure 5:**
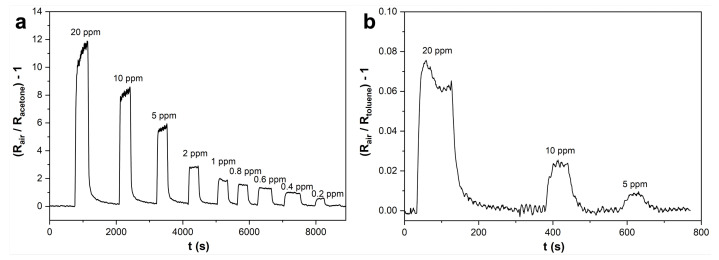
Sensor responses towards decreasing concentrations of (**a**) acetone (20 ppm–200 ppb) and (**b**) toluene (20–5 ppm) gaseous molecules. Tests were performed at 300 °C, in simulated air (80% N_2_–20% O_2_).

A simple sensing test was performed at a high temperature (300 °C), optimal for pristine metal oxide nanomaterials, under simulated air (80% N_2_–20% O_2_), varying the concentration of the target gas until a signal was obtained. The results, shown in Figure 5, highlight that the response of our WO_3_ sensor towards acetone is about two orders of magnitude higher when compared to toluene. This selectivity is usually explained heuristically by considering that acetone is a polar molecule containing a strongly polarized carbon-oxygen bond, while toluene is an aromatic molecule with a modest dipole moment and no particularly reactive functional groups. Hence toluene, differently from acetone, may not undergo any reaction on WO_3_, contributing to the sensor response only through direct charge transfer, as suggested for SnO_2_ by Abokifa et al. [44]. On the other hand, partial and total conversion of aromatic compounds over WO_3_-based catalysts and TiO_2_\WO_3_ photocatalysts have been reported experimentally [45,46], hence a contribution to the sensor response coming from surface reactions cannot be excluded a priori.

Further elaboration of the sensor signals in Figure 5 allows the evaluation of the response and recovery times for both target molecules. Comparable response times are found for the two VOC molecules (around 30 s), while the recovery times differ by approximately a factor of two (40 s for toluene and 90 s for acetone).

### 3.4. Theoretical Results

**Figure 6:**
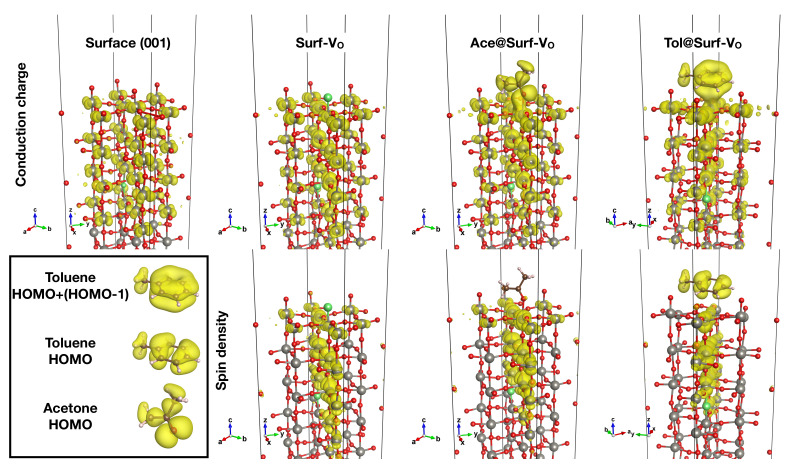
Conduction charge densities (**upper row**) and spin densities (**lower row**) of some of the considered systems. Charge densities of the relevant states of the isolated molecules are reported in the box. The vacancy sites are reported in green.

At the atomic scale, the difference in the sensor responses can be explained in terms of diverse affinities between the target molecule and the surface regulated by: (i) relative size and shape of the molecule and its adsorption site, (ii) electric dipole and overall charge distribution of molecule and surface, (iii) energetic and spatial overlap between the frontier orbitals of the molecules and the electronic states of the surface in proximity of the Fermi level. All these properties were calculated within DFT for a number of systems selected as the most representative of the environment around the WO_3_ surface during the sensing measurements. Their equilibrium structures are shown in Figure 6. In these panels, we also report the conduction charges, i.e., the electron density due to states in the conduction energy region (ρ=0.002 *e*/bohr^3^), along with the corresponding spin density (ρ=0.0005 bohr^−3^). The charge densities of the relevant states of the isolated molecules are reported in the box.

**Figure 7:**
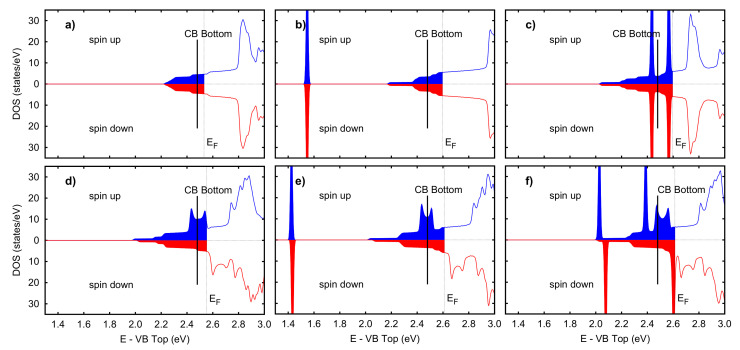
Density of states near the bottom of the conduction bands (CB), for surface and adsorbate systems: (**a**) Stoichiometric surface, (**b**) Ace@Surf, (**c**) Tol@Surf, (**d**) Surf-V_O_, (**e**) Ace@Surf-V_O_, and (**f**) Tol@Surf-V_O_. The black vertical line indicates the position of the bottom of the bulk CB (at 2.46 eV from the top of the valence band). The colored parts mark the occupied states. The broadening of the eigenvalues is 0.01 eV.

The main building block for modeling all the systems investigated herein is the WO_3_ surface (see Figure 4), a slab composed by six WO_6_ octahedra layers where a bulk oxygen vacancy was included. As shown in Figure 7a, the vacancy gives metallic behavior to the system, which is otherwise a wide-gap insulator with a 2.48 eV band gap, according to our GGA-1/2 calculations (see Figure 3). No net spin polarization is observed on this system. We also performed the electron population analysis according to the Quantum Theory of Atoms in Molecules (QTAIM) [47], as implemented in the Critic2 code [48]. It suggests that the two electrons released into the lattice by V_O_ accumulates preferentially into the atomic basins of the first four W layers, though no localized state is present. The terminal oxygen atoms bear on average 0.25 electrons less with respect to the other oxygen atoms, which can be considered as O^2−^.

When a surface oxygen vacancy is introduced in addition to the bulk one (system Surf-V_O_), spin-polarized double-peak structures appear close to the Fermi level (see Figure 7d). This feature is due to the almost one-dimensional and spin-polarized electronic state localized in the surface region and in the plane containing both vacancies (see the second column of Figure 6). In this case, the three W bonded with the terminal oxygens are found in the +5 oxidation state. Due to the cooperative effect of the two vacancies, a tungsten atom near the bulk vacancy reduces to W^5+^. The formation of a surface vacancy does not alter the electronic properties of the three terminal oxygen atoms, i.e., they have about 0.25 electrons less than bulk ones. The global effect is a net magnetic moment of 1.04 $\mu_{B}$ on the system, as depicted in spin density maps. Previous studies pointed towards the presence of surface vacancies on WO_3_, either due to the high reactivity of surface oxygen and their thermally activated desorption [49,50,51].

**Figure 8:**
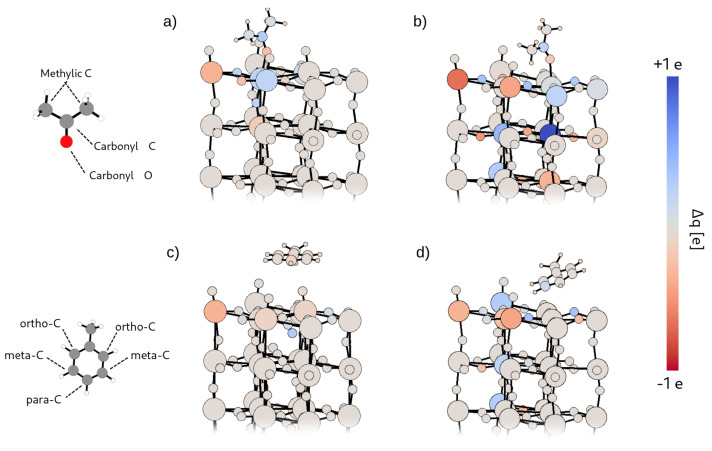
Atomic charge differences color-mapped onto the atomic structures of the systems Ace@Surf (**panel a**), Ace@Surf-V_O_ (**panel b**), Tol@Surf (**panel c**), and Tol@Surf-V_O_ (**panel d**). The charges on the WO_3_ surface are referenced to the ones of the isolated surface, i.e., stoichiometric surface or Surf-V_O_. The charges on the adsorbed molecules are referenced to the ones of the isolated molecule. On the left side are reported the atomic structure of the molecule with labels indicating the chemically different atomic species. The color bar is reported on the right. The same color scale was used for both systems, with an upper and lower limit of 1 electron and −1 electron.

In order to investigate directly their effect on sensing, we simulated acetone adsorption on both a pristine surface and a defective one. In the former case (system Ace@Surf), acetone is strongly adsorbed on the pristine surface with an adsorption energy $E_{\mathrm{ads}}$ of 1.33 eV, which decreases to 1.26 eV when a surface oxygen vacancy is created in proximity of the adsorption site, and further reduces to 0.90 eV when the molecule is adsorbed on top of the vacancy itself (system Ace@Surf-V_O_). This suggests that acetone adsorption is mainly driven by the electrostatic interaction between the negatively charged carbonyl oxygen and the positively charged surface W atoms: as previously discussed, the formation of a surface vacancy produces localized electronic states that reduce the positive charge on the nearby W atoms. This effect is largest on the W atom below the vacancy and justifies the observed trend in the adsorption energies. Figure 7b,e show that both adsorbate systems exhibit a sharp, mid-gap state appearing about 1 eV below the Fermi level, which corresponds to the HOMO state of the isolated molecule. The magnetic moment of Ace@Surf-V_O_ is about 1.0 $\mu_{B}$ while is absent in Ace@Surf. As visualized in Figure 8a,b, the QTAIM atomic charge analysis reveals that acetone’s carbonyl C atom in Ace@Surf and Ace@Surf-V_O_ gains, respectively, 0.33 and 0.25 electrons compared to the isolated molecule. This charge comes partially from the acetone oxygen atom (0.23 *e* for Ace@Surf and 0.18 *e* for Ace@Surf-V_O_). In Ace@Surf, signs of weakening of the C=O bond are also observed, with a 2% increase in length. In all the adsorption configurations, the acetone molecule transfers between 0.20 *e* and 0.27 *e* to the surface—mostly coming from the carbonyl oxygen and from weak electrostatic interactions between the surface and the methylic H—resulting in a partially oxidized state. Overall, the accumulation of negative charge on the carbonyl C, together with the weakening of the C=O bond, can lead to a subsequent breakage of the $sp_{2}$ symmetry on the carbonyl C and activation of a tetrahedral reaction intermediate. This is a typical feature of a carbonyl substitution reaction, which is, in turn, the required initial step for acetone oxidation to CO_2_. Notably, in the case of Ace@Surf-V_O_, we observe a significant negative charge migration towards the bulk, eventually leading to a net 1.0 electron gain for the W atom located below the adsorption site.

Toluene sensing was modeled analogously, including adsorption on both a pristine surface (Tol@Surf) and on a defective one (Tol@Surf-V_O_). The adsorption energetics shows lower binding energies and, interestingly, an opposite trend compared to acetone, with $E_{\mathrm{ads}}$ = 0.17 eV for Tol@Surf and 0.44 eV for Tol@Surf-V_O_. This suggests that toluene, due to its planar geometry and larger size, requires a surface oxygen vacancy in order to have sufficient portion of the surface for a stable adsorption, which is otherwise hindered by the terminal oxygen atoms. The densities of states (see Figure 7c,f) show a non-magnetic state for Tol@Surf, with the two peaks, corresponding to the HOMO and HOMO-1 states of isolated toluene, equally occupied. Differently, for Tol@Surf-V_O_ the two peaks are now spin-polarized—one pinned to the Fermi level, the other ca. 0.2 eV below. Most of the hybridization between the molecule and the surface is observed in the former. The spin-down HOMO is slightly depleted, thus contributing to a total magnetization of 1.16 $\mu_{B}$. The QTAIM atomic charges (see Figure 8c,d) calculated on Tol@Surf-V_O_ indicate that the C atom in *meta*-position to the methyl gains a modest 0.12 electrons compared to the isolated molecule, perfectly balanced by a 0.12 loss on one of the H atoms in *meta*-position. This does not carry significant information about the possible activation of a reactive intermediate, since the reactive carbon atoms for aromatic substitution reactions are notoriously the ones in *ortho*- and *para*-positions. Overall, toluene is in an oxidized state when adsorbed on WO_3_, with a net 0.5 electron loss mostly deriving from weak electrostatic interactions. The charge redistribution induced in the surface slab is modest and less localized as compared to acetone, although showing similar features. This aspect, combined with the large distance between molecule and surface, suggests no reactivity at all for toluene in proximity of a surface region rich in terminal oxygen.

Relevant data about the adsorbate systems are reported in Table 1.

## 4. Conclusions

In this work, we performed sensing tests towards two common volatile organic compounds using a WO_3_ chemiresistor, whose response to acetone is two orders of magnitude higher as compared to toluene. Through ab initio calculations we modeled the WO_3_ surface, the molecules, and the interacting systems, in order to obtain deeper insight about the sensing mechanism and the observed selectivity. Concerning acetone adsorption, our calculated binding energies show a higher affinity with the surface compared to toluene, which translates in a higher probability of effective adsorption and, consequently, higher surface coverage. A QTAIM charge analysis was performed in order to obtain relevant information about the reactivity. The modest overall charge transfer, counterbalanced by a significant reorganization of the electrons in both the molecule’s carbonyl group and the upper surface layers, suggests that the sensor response towards acetone is mainly driven by its oxidation by the surface oxygen rather than a purely electrostatic interaction. Toluene, on the other hand, shows a larger total charge transfer to the surface, but no clear signs of activation towards common aromatic compound reactions. This indicates that toluene oxidation by WO_3_ is less likely to be the main driving force to the sensor response, and a larger contribution from pure charge transfer upon contact with the surface is expected. Interestingly, we observed opposite trends in the adsorption energies of the two molecules when adsorbed on a pristine surface versus a defective one. In particular, surface oxygen vacancies decrease acetone’s affinity for WO_3_ while increasing it for toluene. This suggests that modulating the surface defectivity of the material by acting on the synthesis conditions could be an effective strategy to enhance the selectivity towards acetone (more crystalline surfaces) or, conversely, increasing the sensitivity towards toluene (more defective surfaces). Overall, a selected set of theoretical computations can provide useful information on the chemical interactions underlying the sensing mechanism, and potentially can be extended to the study of any sensor and target molecule.

## Figures and Tables

**Table 1:** Adsorption energies of the considered systems and their spin polarization.

System	$E_{\mathrm{ads}}$ (eV)	Magnetic Moment ($\mu_{B}$)
WO_3_ surface	-	0.00
Surf-V_O_	-	1.04
Ace@Surf	1.334	0.00
Ace@Surf-V_O_	0.899	1.04
Ace@Surf-V_O_^1^	1.255	0.85
Tol@Surf	0.172	0.00
Tol@Surf-V_O_	0.440	1.16

^1^ Adsorption on regular surface W atom.

## Data Availability

The data presented in this study are available on request from the corresponding author.

## References

[1] Moon Y.K., Kim K.B., Jeong S.Y., Lee J.H. (2022). Designing oxide chemiresistors for detecting volatile aromatic compounds: Recent progresses and future perspectives. Chem. Commun..

[2] Pargoletti E., Cappelletti G. (2020). Breakthroughs in the Design of Novel Carbon-Based Metal Oxides Nanocomposites for VOCs Gas Sensing. Nanomaterials.

[3] Tonezzer M., Izidoro S.C., Moraes J.P.A., Dang L.T.T. (2019). Improved Gas Selectivity Based on Carbon Modified SnO_2_ Nanowires. Front. Mater..

[4] Pargoletti E., Hossain U.H., Di Bernardo I., Chen H., Tran-Phu T., Chiarello G.L., Lipton-Duffin J., Pifferi V., Tricoli A., Cappelletti G. (2020). Engineering of SnO_2_–Graphene Oxide Nanoheterojunctions for Selective Room-Temperature Chemical Sensing and Optoelectronic Devices. ACS Appl. Mater. Interfaces.

[5] Bhat P., Naveen Kumar S.K. (2022). Evaluation of IDE-based flexible thin film ZnO sensor for VOC sensing in a custom designed gas chamber at room temperature. J. Mater. Sci. Mater. Electron..

[6] Pargoletti E., Hossain U.H., Di Bernardo I., Chen H., Tran-Phu T., Lipton-Duffin J., Cappelletti G., Tricoli A. (2019). Room-temperature photodetectors and VOC sensors based on graphene oxide–ZnO nano-heterojunctions. Nanoscale.

[7] Americo S., Pargoletti E., Soave R., Cargnoni F., Trioni M.I., Chiarello G.L., Cerrato G., Cappelletti G. (2021). Unveiling the acetone sensing mechanism by WO_3_ chemiresistors through a joint theory-experiment approach. Electrochim. Acta.

[8] Xing X., Zhu Z., Feng D., Du L., Yang D. (2021). The “screening behavior” of lithium: Boosting H_2_S selectivity of WO_3_ nanofibers. J. Hazard. Mater..

[9] Kim H.R., Haensch A., Kim I.D., Barsan N., Weimar U., Lee J.H. (2011). The role of NiO doping in reducing the impact of humidity on the performance of SnO_2_-based gas sensors: Synthesis strategies, and phenomenological and spectroscopic studies. Adv. Funct. Mater..

[10] Kim H.J., Jeong H.M., Kim H.M., Chung J.H., Kang Y.C., Lee J.H. (2014). Enhanced Ethanol Sensing Characteristics of In_2_O_3_-Decorated NiO Hollow Nanostructures via Modulation of Hole Accumulation Layers. ACS Appl. Mater. Interfaces.

[11] Chen Q., Zheng J., Liu X., Zhang X., Kang W., Fang L., Zhou M. (2020). First-principles investigations on the mechanism of highly sensitive and selective trimethylamine sensing in MoO_3_. Appl. Surf. Sci..

[12] Zhang Y., Li D., Qin L., Liu D., Liu Y., Liu F., Song H., Wang Y., Lu G. (2018). Preparation of Au-loaded TiO_2_ pecan-kernel-like and its enhanced toluene sensing performance. Sens. Actuators B Chem..

[13] Pargoletti E., Tricoli A., Pifferi V., Orsini S., Longhi M., Guglielmi V., Cerrato G., Falciola L., Derudi M., Cappelletti G. (2019). An electrochemical outlook upon the gaseous ethanol sensing by graphene oxide-SnO_2_ hybrid materials. Appl. Surf. Sci..

[14] Choi P.G., Kim K., Itoh T., Masuda Y. (2021). Tin Oxide Nanosheets on Microelectromechanical System Devices for Improved Gas Discrimination. ACS Appl. Nano Mater..

[15] Mirzaei A., Kim J.H., Kim H.W., Kim S.S. (2018). Resistive-based gas sensors for detection of benzene, toluene and xylene (BTX) gases: A review. J. Mater. Chem. C.

[16] Kim J.H., Wu P., Kim H.W., Kim S.S. (2016). Highly Selective Sensing of CO, C_6_H_6_, and C_7_H_8_ Gases by Catalytic Functionalization with Metal Nanoparticles. ACS Appl. Mater. Interfaces.

[17] Gong Y., Wu X., Chen J., Li W., Han N., Zhang D., Chen Y. (2019). Enhanced gas-sensing performance of metal@ZnO core–shell nanoparticles towards ppb–ppm level benzene: The role of metal–ZnO hetero-interfaces. New J. Chem..

[18] Li F., Qin Q., Zhang N., Chen C., Sun L., Liu X., Chen Y., Li C., Ruan S. (2017). Improved gas sensing performance with Pd-doped WO_3_·H_2_O nanomaterials for the detection of xylene. Sens. Actuators B Chem..

[19] Suematsu K., Watanabe K., Tou A., Sun Y., Shimanoe K. (2018). Ultraselective Toluene-Gas Sensor: Nanosized Gold Loaded on Zinc Oxide Nanoparticles. Anal. Chem..

[20] Wang J., Su J., Chen H., Zou X., Li G.D. (2018). Oxygen vacancy-rich, Ru-doped In_2_O_3_ ultrathin nanosheets for efficient detection of xylene at low temperature. J. Mater. Chem. C.

[21] Pargoletti E., Verga S., Chiarello G.L., Longhi M., Cerrato G., Giordana A., Cappelletti G. (2020). Exploring Sn_x_Ti_1−x_O_2_ Solid Solutions Grown onto Graphene Oxide (GO) as Selective Toluene Gas Sensors. Nanomaterials.

[22] Song Z., Ye W., Chen Z., Chen Z., Li M., Tang W., Wang C., Wan Z., Poddar S., Wen X. (2021). Wireless Self-Powered High-Performance Integrated Nanostructured-Gas-Sensor Network for Future Smart Homes. ACS Nano.

[23] Perdew J.P., Burke K., Ernzerhof M. (1996). Generalized Gradient Approximation Made Simple. Phys. Rev. Lett..

[24] Soler J.M., Artacho E., Gale J.D., García A., Junquera J., Ordejón P., Sánchez-Portal D. (2002). The SIESTA method for ab initio order-N materials simulation. J. Phys. Condens. Matter.

[25] Troullier N., Martins J.L. (1991). Efficient pseudopotentials for plane-wave calculations. Phys. Rev. B.

[26] Ferreira L.G., Marques M., Teles L.K. (2008). Approximation to density functional theory for the calculation of band gaps of semiconductors. Phys. Rev. B.

[27] Ferreira L.G., Marques M., Teles L.K. (2011). Slater half-occupation technique revisited: The LDA-1/2 and GGA-1/2 approaches for atomic ionization energies and band gaps in semiconductors. AIP Adv..

[28] Vogt T., Woodward P.M., Hunter B.A. (1999). The High-Temperature Phases of WO_3_. J. Solid State Chem..

[29] Woodward P.M., Sleight A.W., Vogt T. (1995). Structure refinement of triclinic tungsten trioxide. J. Phys. Chem. Solids.

[30] Loopstra B.O., Rietveld H.M. (1969). Further refinement of the structure of WO_3_. Acta Crystallogr. B.

[31] Howard C.J., Luca V., Knight K.S. (2002). High-temperature phase transitions in tungsten trioxide—The last word?. J. Phys. Condens. Matter.

[32] Butler M.A. (1977). Photoelectrolysis and physical properties of the semiconducting electrode WO_3_. J. Appl. Phys..

[33] Koffyberg F.P., Dwight K., Wold A. (1979). Interband transitions of semiconducting oxides determined from photoelectrolysis spectra. Solid State Commun..

[34] Miyake K., Kaneko H., Sano M., Suedomi N. (1984). Physical and electrochromic properties of the amorphous and crystalline tungsten oxide thick films prepared under reducing atmosphere. J. Appl. Phys..

[35] González-Borrero P.P., Sato F., Medina A.N., Baesso M.L., Bento A.C., Baldissera G., Persson C., Niklasson G.A., Granqvist C.G., Ferreira da Silva A. (2010). Optical band-gap determination of nanostructured WO_3_ film. Appl. Phys. Lett..

[36] Shen Z., Zhao Z., Wen J., Qian J., Peng Z., Fu X. (2018). Role of Oxygen Vacancies in the Electrical Properties of WO_3−_*_x_* Nano/Microrods with Identical Morphology. J. Nanomater..

[37] Rinaldi F.G., Arutanti O., Arif A.F., Hirano T., Ogi T., Okuyama K. (2018). Correlations between Reduction Degree and Catalytic Properties of WO*_x_* Nanoparticles. ACS Omega.

[38] Lambert-Mauriat C., Oison V. (2006). Density-functional study of oxygen vacancies in monoclinic tungsten oxide. J. Phys. Condens. Matter.

[39] Greiner M.T., Chai L., Helander M.G., Tang W.M., Lu Z.H. (2012). Transition Metal Oxide Work Functions: The Influence of Cation Oxidation State and Oxygen Vacancies. Adv. Func. Mater..

[40] Sachs M., Park J.S., Pastor E., Kafizas A., Wilson A.A., Francàs L., Gul S., Ling M., Blackman C., Yano J. (2019). Effect of oxygen deficiency on the excited state kinetics of WO_3_ and implications for photocatalysis. Chem. Sci..

[41] Oliver P.M., Parker S.C., Egdell R.G., Jones F.H. (1996). Computer simulation of the surface structures of WO_3_. J. Chem. Soc. Faraday Trans..

[42] Wang F., Di Valentin C., Pacchioni G. (2012). DFT Study of Hydrogen Adsorption On the Monoclinic WO_3_ (001) Surface. J. Phys. Chem. C.

[43] Migas D.B., Shaposhnikov V.L., Borisenko V.E., Skorodumova N.V. (2017). The Surface Energy and Band Structure of *γ*-WO_3_ Thin Films. Sci. Adv. Mater..

[44] Abokifa A.A., Haddad K., Fortner J., Lo C.S., Biswas P. (2018). Sensing mechanism of ethanol and acetone at room temperature by SnO_2_ nano-columns synthesized by aerosol routes: Theoretical calculations compared to experimental results. J. Mater. Chem. A.

[45] Li J.J., Weng B., Cai S.C., Chen J., Jia H.P., Xu Y.J. (2018). Efficient promotion of charge transfer and separation in hydrogenated TiO_2_/WO_3_ with rich surface-oxygen-vacancies for photodecomposition of gaseous toluene. J. Hazard. Mater..

[46] Balzer R., Drago V., Schreiner W.H., Probst L.F. (2014). Synthesis and structure-activity relationship of a WO_3_ catalyst for the total oxidation of BTX. J. Braz. Chem. Soc..

[47] Bader R.F.W. (1990). Atoms in Molecules: A Quantum Theory.

[48] Otero-de-la Roza A., Johnson E.R., Luaña V. (2014). Critic2: A program for real-space analysis of quantum chemical interactions in solids. Comput. Phys. Commun..

[49] Li Y., Wang C., Zheng H., Wan F., Yu F., Zhang X., Liu Y. (2017). Surface oxygen vacancies on WO_3_ contributed to enhanced photothermo-synergistic effect. Appl. Surf. Sci..

[50] Gillet M., Lemire C., Gillet E., Aguir K. (2003). The role of surface oxygen vacancies upon WO_3_ conductivity. Surf. Sci..

[51] Liu Y., Li J., Tang H., Li W., Yang Y., Li Y., Chen Q. (2016). Enhanced photoelectrochemical performance of plate-like WO_3_ induced by surface oxygen vacancies. Electrochem. Commun..

